# Developmental characteristics of the permanent upper lateral incisor in unilateral cleft lip and palate

**DOI:** 10.1007/s10006-024-01226-1

**Published:** 2024-02-15

**Authors:** Tim B. A. Knüppe, Mona Haj, Elske M. Strabbing, Eppo B. Wolvius, Paola L. Carvajal Monroy

**Affiliations:** Department of Oral and Maxillofacial Surgery, Special Dental Care and Orthodontics, Erasmus Medical Center, Wytemaweg 80, 3015 CN Rotterdam, The Netherlands

**Keywords:** Humans, Child, Male, Cleft lip, Cleft palate, Incisor, Alveolar bone grafting, Radiography, Panoramic, Tooth abnormalities

## Abstract

**Objectives:**

This study aims to provide insights into the developmental characteristics of the upper lateral incisor in individuals with unilateral clefts.

**Materials and methods:**

Panoramic radiographs of a consistent group of Caucasian children taken over time (ages 6, 9, and 12) were extensively reviewed. The study assessed the distribution pattern, eruption path, tooth development, and crown size of the upper lateral incisor within the cleft region.

**Results:**

The most commonly observed distribution pattern was the lateral incisor located distal to the cleft, accounting for 49.2% of cases. Furthermore, a significant delay in tooth development of the upper lateral incisor on the cleft side was noted at ages 6 and 9 (*p* > 0.001). Compared with the non-cleft side, these incisors often erupted along the alveolar cleft and exhibited microdontia (88.3%, *p* < 0.041).

**Conclusion:**

Lateral incisors on the cleft side display unique distribution patterns, microdontia, and delayed tooth development. Careful monitoring of the cuspid eruption is essential, as it can influence the eruption of the lateral incisor.

**Clinical relevance:**

A comprehensive understanding of the development of the upper lateral incisor relative to the cleft is vital for determining its prognosis over time. The position of the upper lateral incisor can also influence the timing and prognosis of secondary alveolar bone grafting. Preserving the upper lateral incisor favors arch length, perimeter, and symmetry in individuals with unilateral clefts**.**

## Introduction

The upper lateral incisor originates from dental epithelia within the maxillary and medial nasal processes [1]. A failure in the fusion of these processes can result in agenesis or variations in the number and pattern distribution of the upper lateral incisor relative to the alveolar cleft [2, 3]. Moreover, the upper lateral incisor on the cleft side has a substantial developmental delay compared to its counterpart on the non-cleft side [4–6].

The timing of secondary alveolar bone grafting (SABG) can be significantly influenced by the presence, localization, and developmental stage of the lateral incisor on the cleft side [7–9]. The primary goal of SABG is to provide continuity and stability to the upper arch, thereby facilitating the potential eruption of the canine or lateral incisor relative to the cleft area [10]. Notably, preserving the lateral incisor offers additional benefits, including the establishment of adequate arch length, perimeter, and symmetry [11–15]. A comprehensive understanding of cleft-sided lateral incisor development provides new insights into determining the best timing for the SABG procedure.

While numerous studies have investigated the frequency, location, size, and developmental stage of the upper lateral incisor in the cleft area [16–18], limited knowledge exists regarding how the upper lateral incisor develops within the same patient cohort over time. This study examines the development of the upper lateral incisor in a group of individuals affected by non-syndromic unilateral cleft lip and palate at ages 6, 9, and 12.

## Subjects and methods

This study was performed in line with the principles of the Declaration of Helsinki. Ethical approval was obtained by the Medical Ethical Committee of Erasmus Medical Center (C-319726) for the retrospective cohort study.

### Population

The study group consisted of consecutive patients born between 1994 and 2010 (n = 236) referred to the cleft palate team at Erasmus MC – Sophia Children's Hospital (See treatment surgical protocol in Table 1). The inclusion criteria for the study group were: (1) a non-syndromic complete unilateral cleft lip and palate, with a confirmed diagnosis by medical records and neonatal pictures; (2) Caucasian ethnicity; and (3) the availability of orthopantomograms at ages 6, 9, and 12 years. Individuals with incomplete dental records were excluded from the analysis.

**Table 1:** Erasmus MC—Sophia Children's Hospital cleft treatment surgical protocol

Timing (Age)	Procedure
3 months	Lip repair with primary correction of the nasal ala and/or placement of grommets when indicated
9 months	Soft palate closure
9–12 years	Simultaneous hard palate closure* and alveolar bone grafting
18 years	Secondary corrective surgeries e.g., Le fort I, nose, or lip correction

^*^Hard palate closure may be performed at an earlier age if indicated by nasendoscopy findings

### Study procedures

Orthopantomograms at 6, 9, and 12 years underwent comprehensive evaluation by two independent observers (T.K. and P.C.M.) and were recorded using Castor Electronic Data Capture [19]. Data included variables such as age, gender, and cleft side.

### Distribution pattern of the lateral incisor

**Fig. 1:**
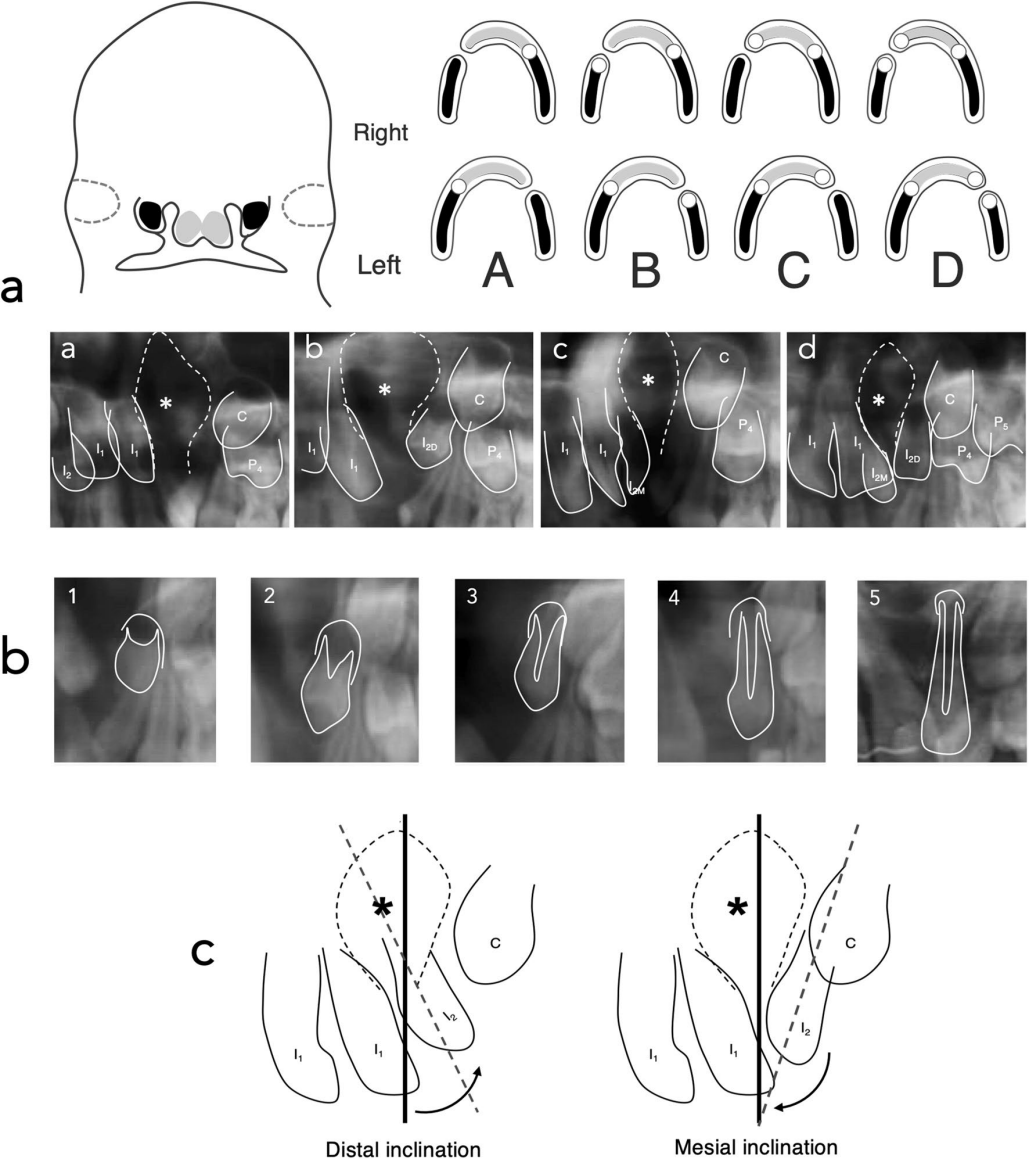
Distribution pattern of the lateral incisor of the upper lateral incisor relative to the alveolar cleft, lateral incisor developmental stages, and axial tooth angulation. Note, a. First row: Development scheme of the human upper jaw and dental arch, the maxillary (black) and medial nasal (light gray) processes. The upper lateral incisor originates from dental epithelia in both processes. Lack of fusion of both processes results in four phenotypes: A: Agenesis resulting from mesenchymal mass deficiency. B and C: one lateral incisor located mesially or distally to the alveolar cleft reflecting the development of only one component of the lateral incisor, and c: duplicated lateral incisors mesially and distally to the cleft resulting from separate development of the dental epithelia of both processes. Second row: radiographs corresponding to four phenotypes on left alveolar clefts at age 6. Abbreviations: I_1_: central incisor, I_2_: lateral incisor, I_2M_: lateral incisor at the mesial side of the cleft, I_2D_: lateral incisor at the distal side of the cleft, C: canine, P_4_: first premolar, P_5_: second premolar, and (*): cleft area. Adapted from [2]. b. Lateral incisor developmental stages. Radiograph and line drawing for the lateral incisor. Adapted from [20]. c. Axial tooth angulation. The degree of tooth angulation was categorized into mesial and distal inclination relative to the midline. Left: distal inclination. Right: mesial inclination. The black line indicates the median line. The broken line indicates the axis of the lateral incisor on the cleft side. Abbreviations: I_1_: central incisor, I_2_: lateral incisor, C: canine, and (*): cleft area

The upper lateral incisor distribution pattern relative to the alveolar cleft was categorized into four phenotypes (A, B, C, D), as illustrated in Fig. 1A.

### Tooth development

The developmental stages of both cleft and non-cleft lateral incisors were assessed in orthopantomograms at ages 6, 9, and 12, following the descriptive criteria established by Demirjian and Levesque (Fig. 1B) [20]. In cases of duplication, both lateral incisors were examined separately, with the non-cleft lateral incisor serving as a control.

### Tooth angulation and eruption path

Axial tooth angulation was documented for cleft-sided lateral incisors with a developmental score of 3 or higher. The angulation was categorized as mesial or distal concerning the midline (Fig. 1C). Angulation data were not considered when orthodontic appliances were in place. No statistical tests were employed; the eruption path was described narratively.

### Tooth size

The size of the lateral incisor on the cleft side was initially assessed by comparing it to its counterpart on the non-cleft area. If the lateral incisor on the cleft side appeared microdontic on the orthopantomogram, further measurements were conducted based on clinical photographs and dental models after the eruption. Microdontia was confirmed if a mesiodistal crown dimension difference of ≥ 0.5 mm was observed. A threshold of ≥ 0.5 mm difference was selected to confirm microdontia because this difference is known to impact smile attractiveness and, in some cases, requires additional treatment to achieve proper occlusion and esthetics after orthodontic treatment [21].

### Statistical analysis

R statistical software version 4.2.0 was used for data analysis [22]. Descriptive statistics summarized cleft phenotypes and affected cleft sides. Data from phenotypes B, C, and D were collected for tooth development, axial tooth angulation, and tooth size assessment of the cleft side lateral incisor, using the non-cleft lateral incisor as a control.

Next, a power analysis for a paired t-test was conducted to assess the adequacy of our sample size, focusing on detecting differences between cleft and non-cleft sides across various age groups. The calculation employed the following parameters: a sample size of 43 pairs (where 'n' denotes the number of pairs), an anticipated medium effect size (d) of 0.5, a significance level (α) of 0.05, and a two-sided alternative hypothesis. Notably, although we initially aimed for a statistical power of 0.8, the analysis revealed a higher power of approximately 89%, indicating a high probability of finding a statistically significant difference in the population.

Two independent observers rated the upper lateral incisor on each radiograph for tooth development across ages 6, 9, and 12. In the case of disagreement, both observers re-evaluated the radiograph to establish a consensus score. The intraclass correlation coefficient (ICC) was calculated using a two-way mixed-effects model with absolute agreement, considering multiple raters (*k* = 2), to evaluate the agreement in scores on tooth development on both cleft and non-cleft sides. This statistical approach was chosen because it accounts for variability among subjects and raters.

Ordinal logistic regression was employed to investigate the potential disparities in lateral incisor development between females (n = 22) and males (n = 63) at ages 6 and 9. Age 12 was excluded as preliminary data suggested that incisor development had nearly completed by this age. The analysis focused solely on the development of lateral incisors in the non-cleft area. The results revealed that males were about 1.58 and 1.42 times less likely to exhibit progress to higher categories of tooth development than females at ages 6 and 9, respectively. These findings suggest a consistent pattern where females show more advanced tooth development than males. Consequently, we excluded the female group from further analysis to maintain data integrity due to the discrepancies in dental maturity between genders.

The normality of the distributions for variables related to tooth development on both the cleft and non-cleft sides across different age groups was evaluated through the Shapiro–Wilk test. Given the non-normal distribution of the data, a Wilcoxon signed-rank test for paired samples was used to analyze the differences in tooth development between lateral incisors located on the mesial and distal sides of the cleft at ages 6, 9, and 12.

Subsequently, a series of Wilcoxon signed-rank tests, with Bonferroni correction applied, were conducted to assess differences in upper lateral incisor development between cleft and non-cleft sides across ages 6, 9, and 12, ensuring statistical rigor. Differences, minimum and maximum values, and the median for tooth development were computed for each age group.

Last, all incisors on the cleft side were consolidated into a single group (*n* = 60) and compared with their paired controls to determine the percentage of microdontic incisors. A McNemar's test was employed to ascertain whether there were significant differences between the pairs.

## Results

### Descriptive statistics

A total of 236 consecutive patients born between 1994 and 2010 were reviewed. After applying the specified inclusion and exclusion criteria, the female (n = 22) group was excluded from the study due to discrepancies in dental maturity between genders. Consequently, a final cohort of 63 male patients was chosen for inclusion in this study.

Moreover, we computed the mean ages and standard deviations (SD) at the time the orthopantomograms were taken, categorizing them into three distinct age groups: 6 years (mean age: 6.03 years; SD: 0.34), 9 years (mean age: 9.01 years; SD: 0.16), and 12 years (mean age: 11.96 years; SD: 0.33). Participants received the bone graft between radiographs taken at 9 and 12 years of age (Table 2).

**Table 2:** Time of alveolar bone grafting versus lateral incisor pattern distribution in cleft area

Phenotype	Time of Alveolar Bone Grafting (Mean ± SD years)
Phenotype A	10.31 ± 0.68
Phenotype B	10.16 ± 1.23
Phenotype C	10.67
Phenotype D	10.23 ± 0.79

Participants received alveolar bone grafting between radiographs taken at ages 9 and 12. The optimal timing for alveolar bone grafting in our clinic was primarily determined by the development of the canine tooth, as evidenced by the relatively low variation in mean ages at grafting across different phenotypes. The standard deviation (SD) was not calculated for Phenotype C due to the limited data (only three cases)

### Distribution pattern of the lateral incisor

Based on the distribution pattern of the upper lateral incisor relative to the alveolar cleft, four distinct phenotypes were identified: phenotype A (*n* = 16), phenotype B (*n* = 31), phenotype C (*n* = 3), and phenotype D (*n* = 13), as summarized in Table 3.

**Table 3:** Distribution pattern, affected side in the total group

	Phenotype A (N, %)	Phenotype B (N, %)	Phenotype C (N, %)	Phenotype D (N, %)	Total group N
	16 (25.4%)	31 (49.2%)	3 (4.8%)	13 (20.6%)	63
Cleft side					
Left	12 (29.3%)	21(51.2%)	1 (2.4%)	7 (17.1%)	41
Right	4 (18.2%)	10 (45.5%)	2 (9.1%)	6 (27.3%)	22

Percentages are calculated based on the Total Group (N) of 63 for Phenotypes A, B, C, and D and separately based on a Total Group (N) of 41 for the Cleft side Left and a Total Group (N) of 22 for Cleft side Right

### Tooth development

No statistically significant differences in tooth development were observed between the mesial and distal sides of the cleft across all age groups (*p* > 0.05). Consequently, all incisors on the cleft side were pooled into a single group for further analysis, resulting in a sample size of *n* = 47. In cases of duplicated incisors, a unified ordinal value was assigned to represent their combined development, with a preference given to the higher rating when the ratings for each incisor indicated different developmental stages. Four cases were excluded from the analysis: one due to insufficient radiograph quality, which impeded the scoring of one lateral incisor, and three others due to the absence of lateral incisors resulting from extractions at ages 9 and 12. Therefore, the final analysis was conducted with a sample size of n = 43. The calculated ICC was 1, demonstrating perfect agreement between the observers after reaching a consensus on the scores.

**Fig. 2:**
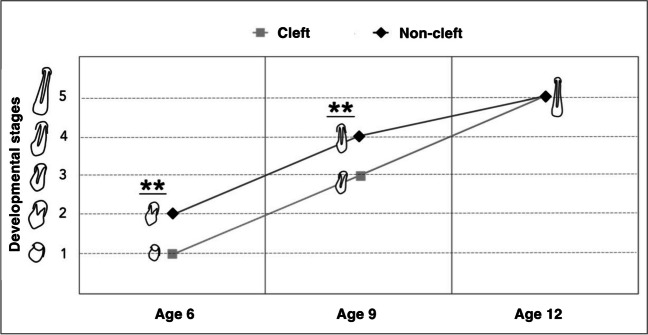
Non-cleft vs. cleft side lateral incisor development. Note: Tooth development was assessed at ages 6, 9, and 12 on the cleft (light gray) and noncleft (black) sides. The figure shows the median at each age point for both groups. Lateral incisor development differs significantly between the cleft and non-cleft sides for at least one developmental stage at ages 6 and 9. Significance: ** (*p* < 0.001). The Wilcoxon signed-rank test with Bonferroni correction was used to calculate *p*-values

**Fig. 3:**
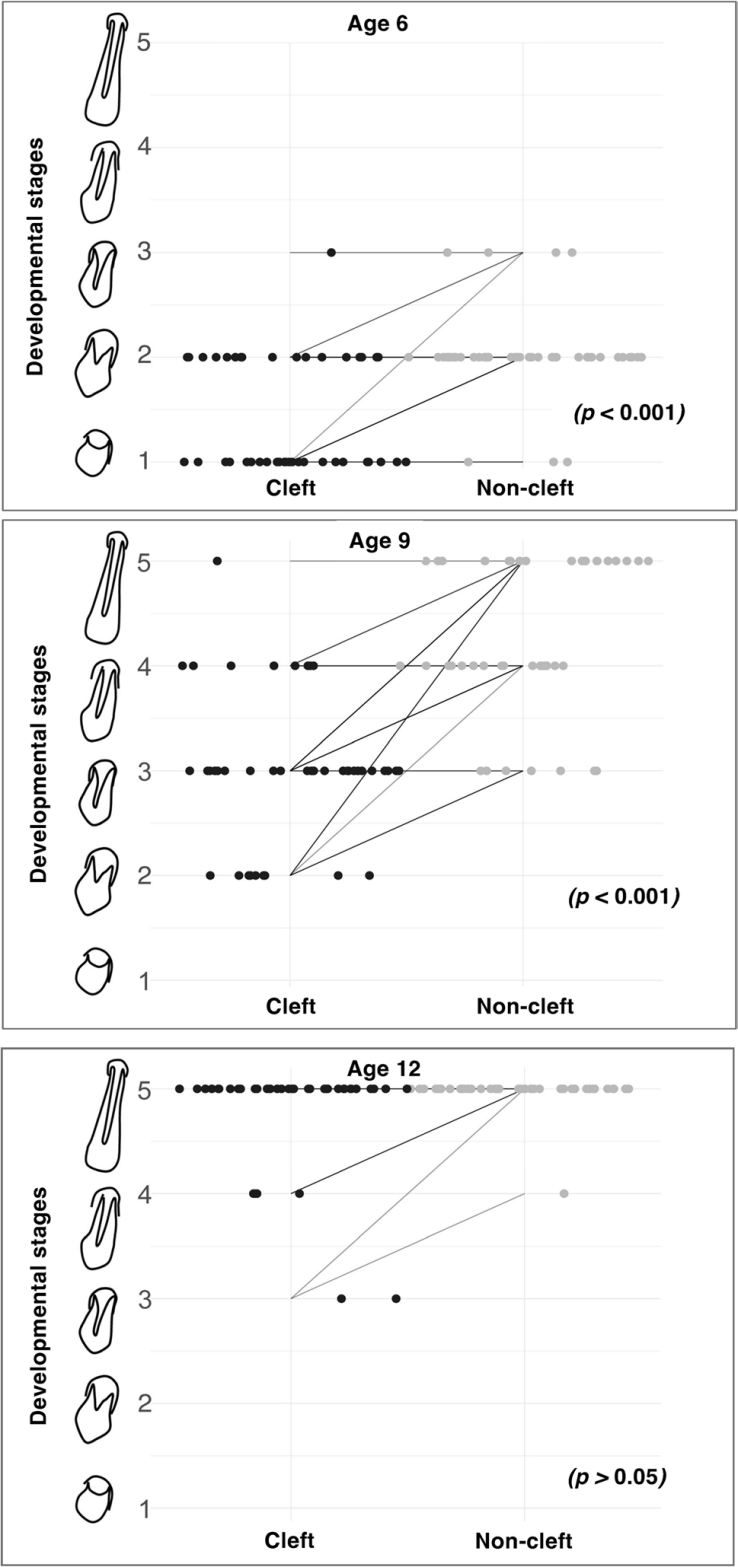
Paired Plot for Tooth Development at Age 6, 9 and 12. Note: The paired plots illustrate the developmental stages of the lateral incisor on both the cleft and non-cleft sides at ages 6, 9, and 12. The dot plots represent data from 43 individual teeth, displaying the progression of tooth development at these stages. At age 6, most teeth are in developmental stage 1, indicating early stages of development. By age 9, increased variation is observed, with most observations showing that over half of the root has formed, reaching stage 3 on the developmental scale. By age 12, most teeth have reached the final stages of development (stages 4 and 5). *p*-values were calculated using the Wilcoxon signed-rank test with Bonferroni correction

Lateral incisor development differed significantly between the cleft and non-cleft sides for at least one developmental stage at ages 6 and 9 (*p* < 0.001, Fig. 2). However, by age 12, no significant differences were observed (*p* > 0.05, Fig. 2), and tooth development had been completed in most cases (Figs. 2 and 3).

### Tooth angulation and eruption path

**Fig. 4:**
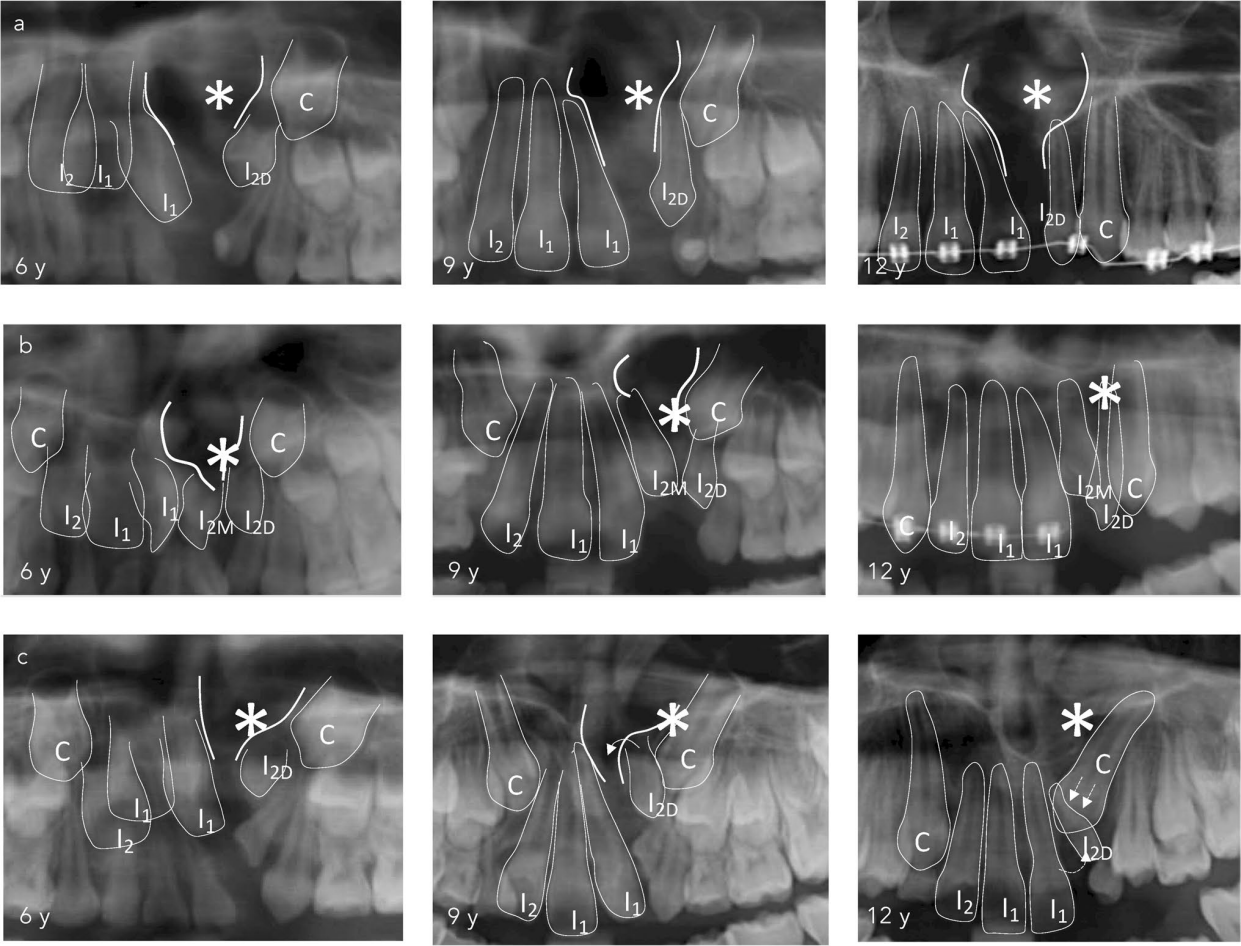
The cleft-side lateral incisor eruption path at ages 6, 9, and 12. Note: representative orthopantomogram imaging provides insights into lateral incisors' eruption trajectory on the cleft side at ages 6, 9, and 12. Abbreviations: I_1_: central incisor, I_2_: lateral incisor, I_2M_: lateral incisor at the mesial side of the cleft, I2_D_: lateral incisor at the distal side of the cleft, C: canine, and (*): cleft area. a. Crown formation of the lateral incisors is typically evident by age 6, and its development and eruption follow the margin of the alveolar cleft. b. Duplicated lateral incisors in the clefted region are typically smaller than the upper lateral incisor in the non-cleft area. Despite one being smaller, no significant differences were observed in the development between both duplicated teeth (*p* > 0,05, Wilcoxon signed-rank test). c. The eruption path of the lateral incisor on the cleft's distal side may change due to pressure from the developing permanent cuspid, causing it to shift into the cleft area

Typically, incisors located distally to the alveolar cleft exhibit a distal inclination following the alveolar cleft, while those located mesially display a mesial inclination relative to the cleft (Fig. 4A and B). However, the eruption path of distally positioned incisors may undergo alterations due to the influence of the developing cuspid pushing its root toward the cleft area (Fig. 4C).

### Tooth size

The results demonstrate a significant prevalence of microdontia among the lateral incisors in the cleft area. Specifically, 88.3% (*n* = 53) of the incisors displayed microdontia, suggesting a greater probability of microdontia on the cleft side (*p* < 0.041).

## Discussion

This study aimed to investigate the developmental characteristics of the upper lateral incisor in a consistent group of individuals with non-syndromic unilateral cleft lip and palate over time. Our findings demonstrate that lateral incisor(s) on the cleft side show variation in pattern distribution, delayed tooth development, and small size.

In alignment with our findings, gender-based disparities in tooth development have been observed in both cleft and non-cleft individuals, with females typically exhibiting more advanced tooth development and earlier dental maturity than males [23, 24]. Consequently, our analysis excluded female individuals due to these disparities. It is critical to acknowledge that including female participants, even in a small group (n = 22), could have influenced the overall conclusions of our study by introducing a bias. Moreover, the restricted sample size limited us to conduct a more comprehensive analysis to provide robust evidence or additional insights into gender-related disparities in dental maturity. Additionally, our study's results are consistent with previous research that has indicated a greater prevalence of clefts on the left side [25].

The most common distribution pattern observed in our study was the location of the lateral incisor distal to the cleft, observed in 49.2% of the cases. This finding contrasts with previous studies, which mainly documented the absence of upper lateral incisors on the cleft side, with prevalence rates ranging from 37.5% to 63% [17, 26]. Our study also found a 20.6% incidence of duplicated upper lateral incisors within the cleft region. These results are similar to previous research, which showed an incidence ranging from 18.2% to 31.3% [4, 26, 27]. Nevertheless, more recent research shows significantly reduced incidence rates, varying between 3.2% and 7.2% [28–31]. The observed disparities in these findings might be attributed to using a cross-sectional design, which inherently collects data at a singular time. Thus, prior procedures, such as dental extractions, can potentially introduce bias into the outcomes. Notably, the distribution patterns seen in clefts are caused by the maxillary and nasal processes not fusing together [2, 3]. Furthermore, a correlation has been identified between agenesis and the extent of the cleft, underscoring the intricate nature of dental development [4].

Consistent with previous studies, the cleft side's lateral incisor(s) showed a delay in tooth development [5, 6, 30, 32–35]. We assessed the development of the upper lateral incisor in a consistent group of children affected by non-syndromic unilateral cleft lip and palate at ages 6, 9, and 12, providing a more comprehensive understanding. Our results indicate that the delay in tooth development is no longer significant at 12, suggesting that the underdevelopment observed at ages 6 and 9 may not necessarily indicate a poor long-term prognosis for the tooth. Various aspects could explain this underdevelopment, including genetic factors and inadequate bone and blood supply [5, 6].

Our study shows the impact of the cuspid on the trajectory of lateral incisor eruption, particularly in cases where the lateral incisor is situated distally from the cleft. The cuspid's in-development might push the lateral incisor's root toward the cleft, compromising its prognosis. Proper eruption of the upper lateral incisor, when present, might have a positive effect on maintaining the long-term arch length, perimeter, and symmetry [7, 13–15].

The lateral incisor on the cleft side has a high prevalence of microdontia (88.3%), consistent with previous studies showing that these teeth frequently exhibit reduced size [36–38]. Hence, employing moderate orthodontic forces is necessary to minimize the risk of excessive stress on the tooth, preventing potential root resorption. This approach ensures tooth preservation and long-term arch stability, positively impacting overall dental health and function and possibly reducing the need for additional interventions [15, 39].

Using paired data in this study reduces the impact of confounding variables and individual differences, strengthening the study's methodology. However, using two-dimensional radiographs to assess the lateral incisors hampered, in some cases, the accurate evaluation of the crown's and root's developmental features and dimensions. Additionally, overlapping anatomical structures could affect the outcomes obtained. Despite these limitations, this study offers novel insights into the developmental characteristics of lateral upper incisors relative to cleft conditions.

In summary, the lateral incisor on the cleft side exhibits numerous distribution patterns, a small size, and a significantly delayed development. A thorough comprehension of the development of the upper lateral incisor within the cleft favors the assessment of the tooth's prognosis. In addition, the localization of the upper lateral incisor may directly influence the timing of the SABG, and its proper eruption contributes to reestablishing and maintaining the arch length, perimeter, and symmetry over time. Further research that includes both genders could benefit from a more detailed examination of gender-related disparities in dental maturity.

## Data Availability

No datasets were generated or analysed during the current study.
